# Study of the Expression of Integrins in Smooth Myocytes of the Gastrointestinal Organs After Space Flight

**DOI:** 10.3390/cells15191730

**Published:** 2026-09-22

**Authors:** Tatyana Samoilenko, Viktoria Shishkina, Svetlana Zolotaryova, Andrey Kostin, Alexander Alekhnovich, Artem Volodkin, Anna Kireeva, Igor Buchwalow, Markus Tiemann, Dmitrii Atiakshin

**Affiliations:** 1 Federal State Budget Educational Institution of Higher Education «N.N. Burdenko Voronezh State Medical University», Ministry of Healthcare of the Russian Federation, 394036 Voronezh, Russia; antailkka@mail.ru (T.S.);; 2 The Research and Educational Resource Center for Immunophenotyping, Digital Spatial Profiling and Ultrastructural Analysis Innovative Technologies, RUDN University, 117198 Moscow, Russiavolodkin-av@rudn.ru (A.V.);; 3 Institute for Hematopathology, Fangdieckstr, 75a, 22547 Hamburg, Germany

**Keywords:** smooth muscle tissue, smooth myocyte, integrin alpha-5, spaceflight, stomach, jejunum, gastrointestinal tract

## Abstract

Spaceflight has crucial effects on living organisms, primarily due to weightlessness, which causes essential morphofunctional changes in the tissues of almost all body systems. Smooth muscle tissue is one of the main system-forming components of the gastrointestinal tract, which makes its study particularly relevant to space gastroenterology. Integrins are cell adhesion receptors expressed in practically all cell types, including smooth muscle cells. By mediating interactions with the extracellular matrix and intercellular signaling, smooth muscle cell integrins actively participate in adaptation to microgravity conditions. However, this aspect of smooth muscle tissue function has not been addressed in space gastroenterology until now. The aim of the study was to investigate the expressions of integrins alpha-5, alpha-2, and alpha-1 expression in smooth myocytes of the gastric and jejunal muscular layer in Mongolian gerbils (*Meriones unguiculatus*) after a 12-day spaceflight, which was part of the Foton M-3 project. We detected a slight increase in integrin alpha-1 expression, a trend toward decreased integrin alpha-2 expression, and a significant decrease in integrin alpha-5 expression in the gastric and jejunal muscularis propria in Mongolian gerbils from the spaceflight group, which may indicate weakened interactions between cells and the extracellular matrix and potentially lead to reduced smooth muscle contractility. Research on the fundamental role of integrins in the development of the digestive system adaptive mechanisms to zero-gravity conditions may be useful in developing preventive measures to mitigate the risk of gastrointestinal smooth muscle hypotrophy and maintain the health of astronauts during their professional activities aboard the ISS.

## 1. Introduction

Research studies involving issues of the body’s adaptation to microgravity remain relevant, since it is impossible to fully simulate Earth’s gravitational loading conditions in the ISS orbit. Particular attention is paid to space gastroenterology, which investigates the functioning of the digestive system (DS) under orbital flight factors, including microgravity. Previous studies conducted during biomedical experiments aboard the ISS and other spacecraft detected essential morphofunctional changes developing in the digestive organs [1,2,3]. Understanding the mechanisms underlying the development of adaptive processes to weightlessness is a key factor in maintaining the health and performance of astronauts during long-term orbital missions, and in developing effective preventive measures.

Smooth muscle tissue (SMT) is a vital structural and functional component of the gastrointestinal tract and, unlike striated muscle, consists of spindle-shaped cells—smooth myocytes (SMCs) having a unique ability to contract slowly and for a long time. The intraorgan connective tissue can be considered a gravity-dependent system, which largely determines the specific morphological and functional changes developing in other organ components during orbital flight. Under the influence of spaceflight factors, a number of significant changes occur at the molecular and cellular levels in the connective tissue sheath surrounding smooth muscle cells [2,4]. A key role in the SMC contractile activity is played by alpha-smooth muscle actin (α-SMA), one of the actin isoforms that ensures the regulation of the contractile process and adaptation to various external factors [5].

Our previous studies of the SMC cytoskeletal structure in the gastrointestinal tract of laboratory animals after spaceflight revealed a significantly decreased expression of alpha-smooth muscle actin [1], which may indicate decreased functional SMT activity. To clarify the mechanisms underlying the gravisensitivity of both smooth myocytes themselves and the mechanisms of interaction of the cell membrane with the extracellular matrix, we studied the expression features of the alpha-1, alpha-2, and alpha-5 integrin systems.

Mechanical forces are critical in the regulation of intracellular signaling pathways and significantly influence cell function in various biological processes. Extracellular mechanical signals are perceived by cellular receptors located on the cell surface. These signals are then transmitted into the cell, leading to cytoskeletal remodeling and changes in the interactions between cells and the extracellular matrix. These processes ultimately contribute to the regulation of SMC contractility and remodeling [6]. Integrins, acting as mechanotransducers, mediate the transmission of mechanical forces between the cell and the extracellular matrix. Integrins are proteins located on the surface of cell membranes of a number of cells, including smooth muscle cells, consisting of two types of subunits: alpha (α) and beta (β); they form heterodimers that are of importance in the formation of intercellular contacts and interaction with the extracellular matrix of connective tissue, participate in signaling and affect various cell functions, such as migration, differentiation, proliferation and cell death [7]. When binding to components of the extracellular matrix, integrins initiate intracellular signaling and regulate various functions of smooth muscle cells of the gastrointestinal tract, including proliferation, and cell migration; they are relevant in cell adhesion and in changing the cell phenotype [8,9]. It is well known that the α5β1 protein is closely related to the functional state of smooth muscle cells, in particular with smooth muscle actin and the amount of induced contraction. While α1β1 serves as a receptor for extracellular matrix proteins collagen IV and laminin, and also ensures cell adhesion, proliferation and mobility by modulating the actin cytoskeleton, and integrin α2β1 is the main collagen smooth muscle cell receptor [10,11,12,13]. Furthermore, the possible influence of integrin α5β1 on smooth muscle cell apoptosis is noteworthy [14]. Thus, integrins may be involved in adaptive changes in smooth muscle tissue and the extracellular matrix surrounding them in the digestive system under spaceflight conditions, primarily driven by a reduction in the morphological substrate of contractile activity—the thickness of the smooth muscle layer—as we have demonstrated in previous experiments on various species of laboratory rodents [1,2]. Additionally, integrin α5β1 may participate in the remodeling of the extracellular matrix, including after traumatic injury [15,16], which is also relevant to space biology and medicine. Despite the fact that integrins bear a significant functional load in the SMC contractile activity of the gastrointestinal tract, studies in space biomedicine have not previously been conducted.

## 2. Materials and Methods

### 2.1. Experimental Design

The experimental part of the work was conducted at the State Scientific Center of the Russian Federation Institute of Biomedical Problems of the Russian Academy of Sciences (SSC RF–IBMP RAS). Detailed information on the experiment is presented in articles [17,18]. The study involved three groups of male Mongolian gerbils (*Meriones unguiculatus*). Group 1 consisted of 12 Mongolian gerbils that spent 12 days in orbit aboard the Foton M-3 spacecraft. The animals were withdrawn from the experiment 21 h after returning from spaceflight. Group 2 consisted of 11 Mongolian gerbils from a synchronous ground experiment that were kept for 12 days in the Kontur-L apparatus, a device simulating spaceflight conditions. Gerbils from the synchronous ground-based experiment (11 rodents) were kept for 12 days in a model simulating space flight conditions in the Kontur-L equipment. According to the telemetry results, all the main conditions of space flight were reproduced in this group of experiments, except for weightlessness [17]. Group 3 consisted of 12 animals from a vivarium control that were kept under standard conditions. The morphological part of the work and analysis of the expression level of integrin alpha-5 in smooth myocytes of the gastrointestinal tract after spaceflight were conducted at the Research Institute of Experimental Biology and Medicine, N. N. Burdenko Voronezh State Medical University, using the material from a flight experiment that was part of the Foton M-3 project, a 12-day orbital flight of a spacecraft.

The experimental study was conducted in compliance with the requirements for the humane treatment of animals in accordance with the decision of the Biomedical Ethics Committee of the Russian Research Center IMBP RAS (Protocol No. 206 dated 7 September 2007). The protocol is a fundamental document that sets out the ethical standards and applies, without exception, to all procedures involving live animals conducted within the framework of the spaceflight research project.

### 2.2. Histoprocessing and Tissue Probe Staining

After the animals were removed from the experiment, the preparation of tissue samples for histological and immunohistological examination was carried out according to the following algorithm: fragments of the fundus of the stomach and jejunum of the Mongolian gerbils were carefully removed to avoid crushing or tearing of the tissues, and the samples were immediately fixed in 10% buffered formalin, with the fixator volume exceeding the volume of the organ fragments by at least 10 times [4,19]. After fixation, the samples were processed in a standard manner for no more than two days, which included dehydration, degreasing, and paraffin embedding of the tissues using a MTP-120 histoprocessor (Bochum, Germany), followed by embedding in paraffin blocks using a Tissue-Tec-5 modular embedding station (Sakura, Japan). The sections were cut to a thickness of no more than 4 μm using an Accu-Cut SRM 200 microtome (Sakura, Japan), Gill’s hematoxylin (ErgoProduction LLC, St. Petersburg, Russia) and eosin H “ChDA” (Russia) were used for staining to identify all cellular elements and some non-cellular structures. Immunohistological detection of integrin alpha-5 was performed on 2 μm-thick sections using rabbit monoclonal antibodies against Integrin alpha-5 [EPR7854] ab150361, diluted 1:400 (Abcam, Cambridge, UK). Immunohistochemical detection of integrin alpha-1 was performed using mouse monoclonal antibodies Integrin alpha-1 [CL7207] ab243032, diluted 1:1000 (Abcam, UK); detection of integrin alpha-2 was performed using rabbit monoclonal antibodies Integrin alpha-2 [EPR17338]—Low endotoxin, Azide free ab222377, diluted 1:500 (Abcam, UK). To detect the secondary antibodies, a set of reagents called “Universal Two-Step Detection System PrimeVision” (antibodies to mouse/rabbit IgG—HRP/Dab, PrimeBioMed, Moscow, Russia) was used. Before covering the sections with a cover glass, the nuclei of the microslides were stained with Mayer’s hematoxylin (ErgoProduction LLC, St. Petersburg, Russia). For the negative control, the samples of the studied sections were subjected to a standard immunohistochemical procedure without incubation with primary antibodies. After the animals were withdrawn from the experiment, fragments of the rodents’ stomach and jejunum were fixed in 10% formalin according to the standard sample preparation protocol.

### 2.3. Image Acquisition

Microscopic specimens were analyzed and photographically documented across 25 fields of view using a ZEISS Axio Imager.A2 microscope (Göttingen, Germany) with a ×100 objective (Zeiss alpha Plan-Apochromat objective 100×/1.46 OilDICM27). The immunopositive area was analyzed using ImageJ 1.51J8 software, and the result was expressed as a percentage of the total area of the analyzed image.

### 2.4. Statistical Analysis

The study results were processed using LibreOffice Calc and SPSS Statistics 23.0. The normality of the variation distribution was assessed using the Kolmogorov–Smirnov test. All selected parameters were normally distributed. Statistical hypotheses were evaluated using the parametric Student’s *t*-test for relative values of unrelated samples. Quantitative data in the study were presented as % ± standard error. Differences were considered statistically significant at the significance level *p* < 0.05.

### 2.5. Study Limitations

Since our study was based solely on an immunohistochemical analysis of archived fixed tissue samples obtained earlier from a spaceflight experiment, which are of unique value and are carefully preserved in compliance with all applicable regulations, we did not perform cross-validation using different methods or functional experimental data obtained in animals.

## 3. Results

After staining with Gill’s hematoxylin and eosin, the analyzed microscopic sections of fragments of the gastric fundus manifested morphological changes in the muscular membrane structure in animals from Group 1 (Figure 1C). These changes may indicate the influence of spaceflight factors on the structural tissue organization in the absence of gravitational load [1]. Under physiologically standard conditions, the stomach wall of Mongolian gerbils has a typical histological structure, including the mucosa, submucosa, and three-layer muscular and serous membrane.

Immunohistochemical analysis of integrins α 1, α 2, and α 5 showed that changes in integrin expression under microgravity conditions are cell-specific and depend on the particular subunit. Expression of integrin α 1 exhibited a slight tendency to increase compared to the vivarium control group (Figure 1G–I), whereas integrin α 2 tended to decrease, indicating an adaptive nature of the changes (Figure 1J–L). The divergent reactivity of α 1 and α 2 integrin expression may be associated with the specificities of fibrillogenesis in altered gravity conditions. In a study carried out by Shishkina V. (2023) [2], the histoarchitecture of connective tissue fibers in the small intestine and stomach of mice was examined after a 30 day spaceflight. According to the authors’ findings, the amount of type III collagen around the smooth muscle cell membrane was significantly reduced under conditions of weightlessness [2]. A study of the immunopositive area of alpha-5 integrin expression revealed significant differences between the experimental groups. To improve the accuracy and objectivity of the analyzed results, the slides were examined under high-magnification microscopy using an x100 objective. The immunopositive area of alpha-5 integrin expression in the muscular membrane of the gastric fundus of Mongolian gerbils (*Meriones unguiculatus*) from the control group was 10.79 ± 0.16% of the total area within the analyzed visual fields. Integrin expression in SMCs was characterized by a uniform phenotype, appearing as small bands uniformly distributed across the entire membrane (Figure 1D).

In the analyzed gastric muscular membrane of animals from Group 1, more pronounced strands of connective tissue between the fibers of the muscle tissue were visualized, while in animals from Group 3 (the control group, Figure 1A), the endomysium was characterized by a more ordered and compact structure.

In the studied microscopic specimens of the muscularis propria structure, no differences were found in animals from Group 2 compared to animals from Group 3 (the vivarium control group). Hematoxylin and eosin staining revealed no morphological changes in the structure of the proper smooth muscle cells in animals from all groups (VC, SF, and SE). A similar morphological pattern was also observed in the SMT structure of the jejunal muscular membrane.

In the studied microsections of animals from the synchronous ground experiment group, the immunopositive area was 9.09 ± 0.11% (t = 1.08, *p* > 0.05), exhibiting no significant changes compared to animals of the control group (Figure 1E and Figure 2). In microsections of the gastric muscular membrane fragments of animals from the spaceflight group, a significantly decreased immunopositive area of integrin alpha-5 to 2.46 ± 0.8% (t = 3.94, *p* < 0.05) was revealed. The distribution and intensity of expression were not uniform on the surface of the cell membrane of smooth myocytes; expression was exhibited in the form of intermittent lines, and in some myocytes none expression was visualized. Areas of membranes without immunopositive manifestations of integrin alpha-5 were identified (Figure 1F).

A similar reaction was also observed in the jejunal muscular layer of Mongolian gerbils (*Meriones unguiculatus*). Thus, in the control samples, the immunopositive area of integrin alpha-5 was 8.89 ± 0.15% with a uniform distribution structure over the entire surface of the SMC membrane. In samples of animals from the synchronous experiment group, the percentage was 7.97 ± 0.14% (t = 0.43, *p* > 0.05), and in samples of animals from the 12-day spaceflight experiment group, the percentage of integrin alpha-5 expression was 2.02 ± 0.07% (t = 3.18, *p* < 0.05), demonstrating a significantly decreased parameter compared to the control group. All data are presented in the graph (Figure 2). As a result of the study of smooth muscle actin, a significant decrease in the immune-positive area of α-SMA in the muscle membrane of the fundal stomach of Mongolian gerbils *Meriones unguiculatus* was found in the spaceflight group, where this indicator was 75.80% of the total analyzed area compared to animals from the vivarium control group—90.16%.

In the synchronous experiment group, the percentage of α-SMA expression was 86.23% compared to the vivarium control, showing a slight decrease in this indicator (Figure 3). A similar expression pattern of α-SMA was found in the jejunum muscle membrane. In the spaceflight group, this indicator was 75.71%, showing a significant decrease in the immunopositive area in comparison to the vivarium maintenance group, where the area of α–SMA was 88.97% of the total analyzed area. In the synchronous experiment, it was 83.29% (Figure 3).

Morphological analysis of α-SMA expression in the muscular membranes of the stomach and jejunum of the Mongolian gerbils *Meriones unguiculatus* revealed the following differences between the groups: in the vivarium-containing groups, uniform α-SMA expression was observed. A significant number of cells weakly expressing α-SMA were found in the spaceflight group (Figure 4).

Immunopositive α-SMA cells were surrounded by endomysium (Figure 4A), the fibrous component of connective tissue, corresponding with the morphological norm of the organ structure. The endomysium accounts for approximately 10% of the total area of the muscle membrane. This component does not express α-SMA, which is also normal since there are nerve nodes in the intermuscular stroma of the membrane, the neurons of which do not express α-SMA.

Differentiation of α-SMA-negative smooth myocytes was carried out according to the following morphological features: fusiform shape, and an elongated, rod-shaped centrally localized nucleus (Figure 4). Mainly, areas of smooth myocytes weakly expressing α-SMA were identified at the boundary of two multidirectional layers of SMT: an outer longitudinal layer and an inner circular layer (Figure 4B).

A decrease in the α-SMA immunopositive area in the muscular lining of the stomach and jejunum of the Mongolian gerbil (*Meriones unguiculatus)* can be evaluated as changes in the functional activity of smooth myocytes during spaceflight and could suggest a tendency toward the weakening of motor and peristaltic functions, potentially leading to changes in organ activity.

## 4. Discussion

Our data demonstrate that changes in integrin expression exhibit pronounced cell-specific and subunit-dependent patterns, indicating a complex, multidirectional adaptive response to the absence of gravitational stimulation. In particular, we observed a slight increase in integrin alpha-1 expression, a trend toward decreased integrin alpha-2 expression, and a significant decrease in integrin alpha-5 expression compared to the vivarium control group. This redistribution of the smooth muscle cell receptor apparatus (with a tendency toward the predominance of integrin alpha-1 over integrin alpha-2) fundamentally alters their adhesive interactions with the extracellular matrix. Given that integrin α2β1 is the primary receptor for fibrillar collagens (including type III collagen), whereas integrin α1β1 preferentially binds to type IV collagen and laminin, such a shift in receptor engagement weakens the anchoring of cells to the three-dimensional collagen fiber network.

Connective tissue histoarchitectonic analysis data obtained by the authors of [2] support this hypothesis. According to their findings, under microgravity conditions there is a significant reduction in the type III collagen content in the pericellular space surrounding the smooth muscle cell membrane. Type III collagen serves as a system-forming matrix that establishes the integrative-buffer metabolic environment, which is essential for mechanotransduction, nutrition, and the maintenance of the structural integrity of the muscle wall.

Thus, we observe a direct coupling of molecular and structural changes: decreased expression of integrin alpha-2 (the collagen receptor) correlates with a loss of its primary ligand (type III collagen) in the immediate vicinity of the sarcolemma. This leads to a decoupling of signaling pathways dependent on mechanosensitive integrins, likely triggering a cascade of extracellular matrix remodeling with the loss of its buffer and supportive properties.

The obtained data have fundamental significance for space biomedicine, as they reveal one of the key mechanisms of smooth muscle maladaptation in internal organs—the loss of functional unity between the smooth muscle cell and its connective tissue microenvironment under altered gravitational stimulation.

The study demonstrated a significant decrease in alpha-5 integrin expression in gastric and jejunal smooth muscle cells of Mongolian gerbils (*Meriones unguiculatus*) (the Photon (Foton) M-3) from the 12-day spaceflight group compared to samples of animals from the control group. The decrease in integrin alpha-5 expression in the absence of significant changes in integrin alpha-1 and alpha-2 levels suggests a selective blockade of specific signaling cascades and confirms the differential gravisensitivity of various integrins under spaceflight conditions. A key finding supporting this specificity is that the integrin alpha-5 expression pattern in the synchronous experiment (SE) group was similar to that of the vivarium control (VC) group and differed significantly from the spaceflight (SF) group. This similarity between the VC and SE groups suggests that the significant reduction in integrin alpha-5 expression is specifically attributable to the absence of gravitational loading (microgravity), rather than to other concomitant spaceflight factors that were present in the SE group, such as space restriction, dietary changes, or psychological stress. Based on these results, we hypothesize that integrin alpha-5, acting as a primary mechanotransducer, requires a constant gravitational tension vector to maintain basal expression levels and functional assembly into focal adhesions. In the SE group, all other external stress factors except microgravity were present and provided sufficient mechanical loading to preserve integrin–cytoskeleton coupling. This underscores the exceptional sensitivity of the integrin alpha-5 complex specifically to mechanical unloading, rather than to general physiological stress. Consequently, the imbalance in mechanical signal transmission that arises under microgravity conditions leads to cytoskeletal remodeling in smooth muscle cells, reduced expression of contractile proteins (α-SMA), and the weakening of cell–extracellular matrix interactions. This cascade of events ultimately increases the risk of gastrointestinal motor dysfunction due to impaired smooth muscle contractility. These data may indicate a weakened interaction between cells and the extracellular matrix of the connective tissue, potentially leading to a decrease in SMC contractility.

Lin X. et al. [20] reported that factors of spaceflight can have a significant influence on the properties of cell adhesion in laboratory animals in experiments. Cell adhesion is divided into adhesion between cells and between cells and the extracellular matrix. These processes trigger recognition between cells, as well as conjugation, the process of migration, remodeling of the cytoskeleton and signal transmission. Structural changes and restructuring of the cytoskeleton are the main reason for changes in cell adhesion [20].

It is known that the cytoplasmic tails of the β-subunit of integrin α5 (particularly β1, β3) serve as a platform for the assembly of focal adhesions, which involve talin (a key adaptor linking the integrin β-tail to actin filaments), vinculin, α-actinin, zyxin, and paxillin (additional proteins that stabilize the integrin–cytoskeleton connection), as well as a number of signaling proteins. As a result, the Integrin Adhesion Complex (IAC) is formed, which physically connects the extracellular matrix via integrins to the actin microfilaments (stress fibers) of smooth muscle cells. It is evident that the reduced expression of integrin α5β1 may lead to impaired functioning of the IAC and, consequently, to a weakening of the contractile efficiency of smooth muscle cells under microgravity conditions, because only as part of a functional multicellular sheet can smooth muscle cell contraction effectively change the lumen of hollow organs, including those of the digestive system. Thus, a decrease in the integrin α5β1 content may lead to distorted peristaltic activity of both the stomach and the intestines during spaceflight. Integrin is involved in the mechanotransduction pathway, connecting extracellular matrix components to the actin cytoskeleton with the participation of talin and vinculin. Tension can induce conformational changes in the adhesion complex, recruiting additional proteins into the complex in proportion to the applied force, consistent with the concept of a ‘catch bond’ [21,22]. Compared to skeletal muscle, smooth muscle cells possess high mechanical plasticity and can rapidly remodel their focal adhesions in response to changes in load [23]. This mechanism is necessary for maintaining basal tone, which is mediated by integrin in close association with talin and vinculin. Reduced integrin expression under microgravity conditions may impair the mechanosensitivity not only of individual smooth muscle cells but also of the entire muscle layer, consequently provoking changes in the peristalsis of hollow organs of the digestive system [21,22]. Moreover, since in smooth muscle cells integrins are localized in caveolar membrane domains that act as ‘rapid response’ mechanosensors, reduced integrin levels may disrupt their mechanosensitivity. Furthermore, the involvement of integrins in transmitting mechanical tension via the actin cytoskeleton to the nuclear envelope through LINC complexes (nesprin–SUN) provides a basis for effects on gene expression. In this case, in response to mechanical stimuli, integrins, through activation of transcription of certain cofactors such as YAP or TAZ, may enhance the biogenesis of protein components of the smooth muscle contractile apparatus [21,22]. Thus, the decreased integrin α5β1 content may be the cause of the reduced smooth muscle actin expression we observed, affecting the gravisensitivity of nuclear mechanotransduction.

The results of our studies on the effects of spaceflight factors on the digestive system in laboratory animals showed a significant decrease in the expression of α-SMA in smooth myocytes in the spaceflight group [1]. As previously described, integrins play a key role in forming a connection between the extracellular matrix and the actin cytoskeleton, promoting cell adhesion. The restructuring of the SMC cytoskeleton in the study led to a decrease in cellular mechanosensitivity due to changes in cell adhesion to the extracellular matrix of connective tissue. The incomplete assembly of cell adhesions creates a feedback loop for signal transmission through integrins, which contributes to further restructuring of the actin cytoskeleton [6].

Extracellular mechanical signals are perceived by cell receptors located on the cell surface. These signals are then transmitted into the cell, leading to a restructuring of the cytoskeleton and changes in the interactions between cells and the extracellular matrix. These processes ultimately contribute to the regulation of contractility, the remodeling of smooth muscle cells, and also cell proliferation [24]. Integrins, acting as mechano-transducers, transmit mechanical forces between the cell and the extracellular matrix by interacting with the cytoskeleton and α-SMA through dense bodies.

It is hypothesized that the gravity-dependent reduction in α-SMA expression in microgravity conditions may lead to the disruption of the actin cytoskeleton, which is considered an intracellular effector that responds to mechanical stimuli from transmembrane integrin complexes [25].

Understanding the mechanisms associated with integrins is of particular importance not only for molecular morphology, but also for practical applications, such as developing new therapeutic approaches to the treatment of diseases resulting from disorders in the SMT function [26], and introducing new preventive measures to maintain the health of astronauts. Research studies related to changes in the expression of integrins under spaceflight conditions are extremely limited [27]. The data obtained by Gridley et al. [28] indicate a complex body response to the effects of spaceflight affecting various systems. A detailed study of gene expression in the lungs of mice exposed to long-term flight conditions revealed changes associated with the extracellular matrix, cellular adhesion and intercellular signaling. The increased expression of genes, including integrin alpha-5, detected in the study, as well as the apoptosis observed in the lungs of mice in the experiment, serve as additional evidence of the significant impact of spaceflight conditions on the body [28].

Beyond its relevance to space biomedicine, the observed downregulation of integrin alpha-5 and α-SMA has important implications for understanding clinical pathological conditions characterized by smooth muscle dysfunction. In the gastrointestinal tract, a similar phenotypic shift—marked by reduced α-SMA levels and altered integrin-mediated adhesion—is a hallmark of motor disorders such as gastroparesis, intestinal pseudo-obstruction, and structural tissue remodeling [29,30]. Reduced integrin alpha-5 expression impairs mechanotransduction, which not only compromises contractility but may also trigger a phenotypic switch of smooth muscle cells from a contractile to a synthetic state. In this state, cells lose specialized contractile proteins and acquire the capacity for active proliferation and extracellular matrix production. This synthetic phenotype plays a significant role in the pathogenesis of vascular diseases as well as in the progression of gastrointestinal fibrosis [31,32]. Thus, our data suggest that the removal of gravitational loading serves as a valuable experimental model for studying the early molecular mechanisms of smooth muscle cell dedifferentiation and extracellular matrix remodeling, opening up potential therapeutic targets for diseases associated with smooth muscle atrophy.

The results of the study demonstrate that the gastrointestinal tract SMT exhibits a specific response to stress factors that arise during spaceflight. In the course of the study, through the identified structural rearrangements of the actin cytoskeleton of smooth myocytes, which led to a cascade of reactions such as the incomplete assembly of cell adhesion complexes and the formation of a feedback loop for the transmission of signaling molecules through integrins, it was established that integrins can act as central mediators that link mechanical stimuli to intracellular signaling pathways. Additional research is required to further understand their role in the adaptation of the SMT to microgravity conditions, focusing on the molecular mechanisms of integrin-mediated signaling. Thus, integrins not only ensure the structural integrity of the SMT, but are also important proteins that regulate cell adaptation to environmental changes, making them a subject of research in the field of space medicine.

## Figures and Tables

**Figure 1:**
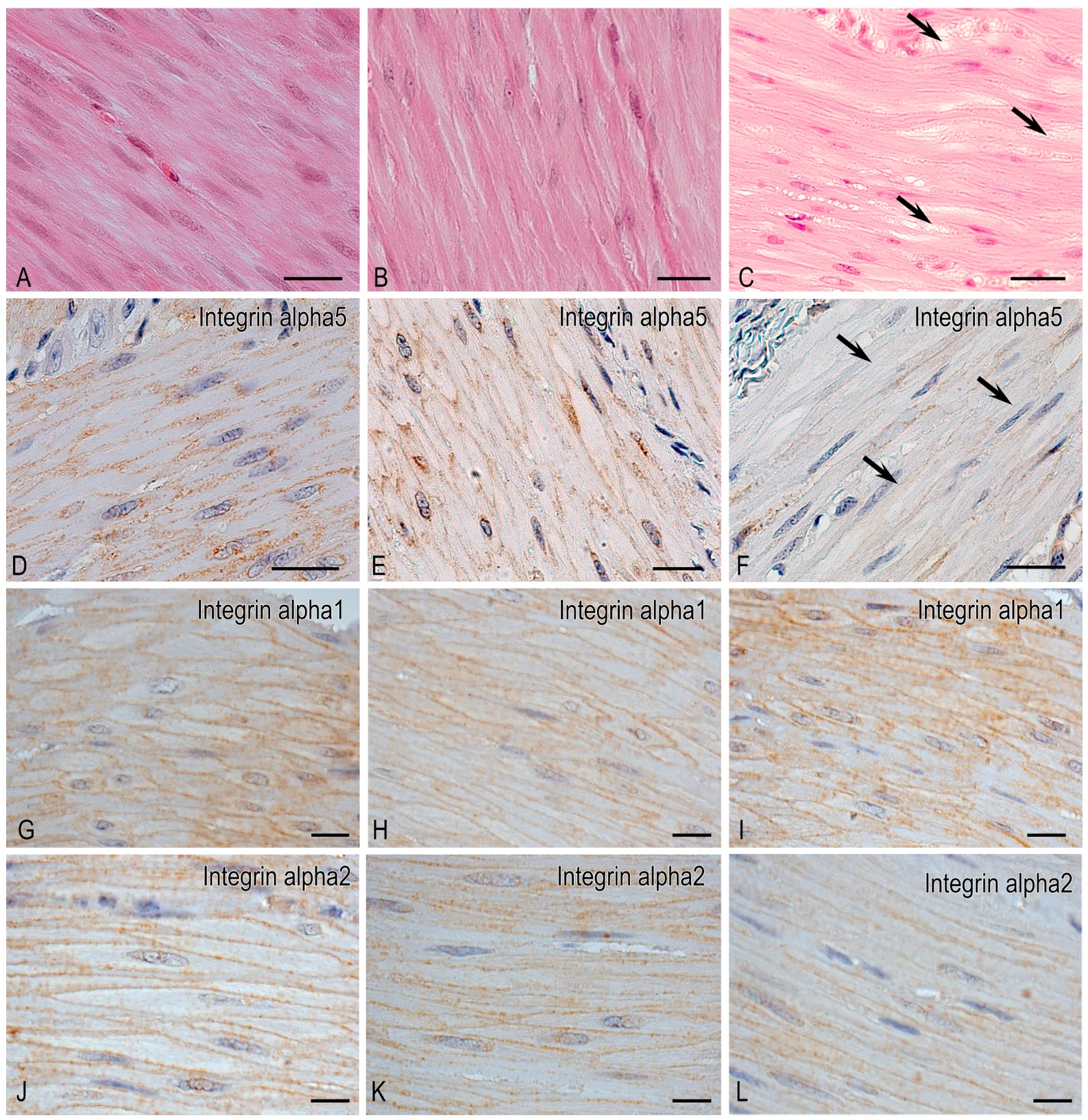
Fragments of the gastric fundus of Mongolian gerbils. Techniques: (**A**–**C**)—histological staining with Gill’s hematoxylin and eosin; (**D**–**F**)—immunohistochemical detection of integrin alpha-5 (**D**–**F**), integrin alpha-1 (**G**–**I**), and integrin alpha-2 (**J**–**L**). (**A**,**D**,**G**,**H**)—vivarium control (Group 3); (**B**,**E**,**H**,**K**)—synchronous ground experiment (Group 2); (**C**,**F**,**I**,**L**)—spaceflight experiment (Group 1). Scale—10 µm. Notes: In (**C**), the arrows indicate connective tissue strands; in (**F**), the arrows indicate immunonegative areas of the muscular membrane, smooth myocytes that do not express or weakly.

**Figure 2:**
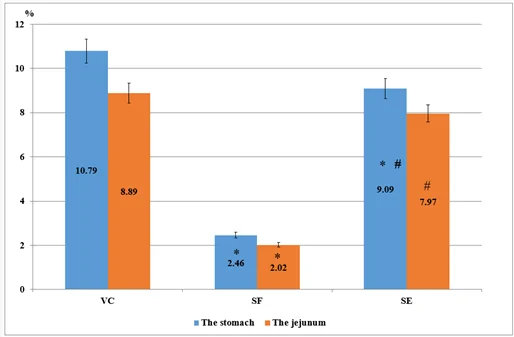
Diagram of alpha-5 integrin expression in the muscularis propria of the stomach and jejunum of Mongolian gerbils. Note: **VC**—vivarium control, **SF**—space flight, **SE**—synchronous ground experiment. *—statistically significant change compared with VC; #—statistically significant difference compared with SF.

**Figure 3:**
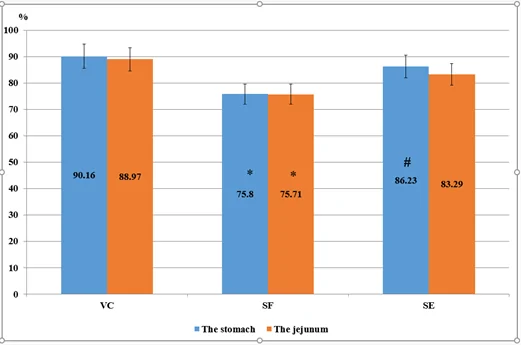
The level of the immunopositive area of α-SMA in the muscular membranes of the stomach and jejunum of Mongolian gerbils. Note: **VC**—vivarium control, **SF**—spaceflight, **SE**—synchronous ground experiment. *—statistically significant difference compared with VC; #—statistically significant difference compared with SF.

**Figure 4:**
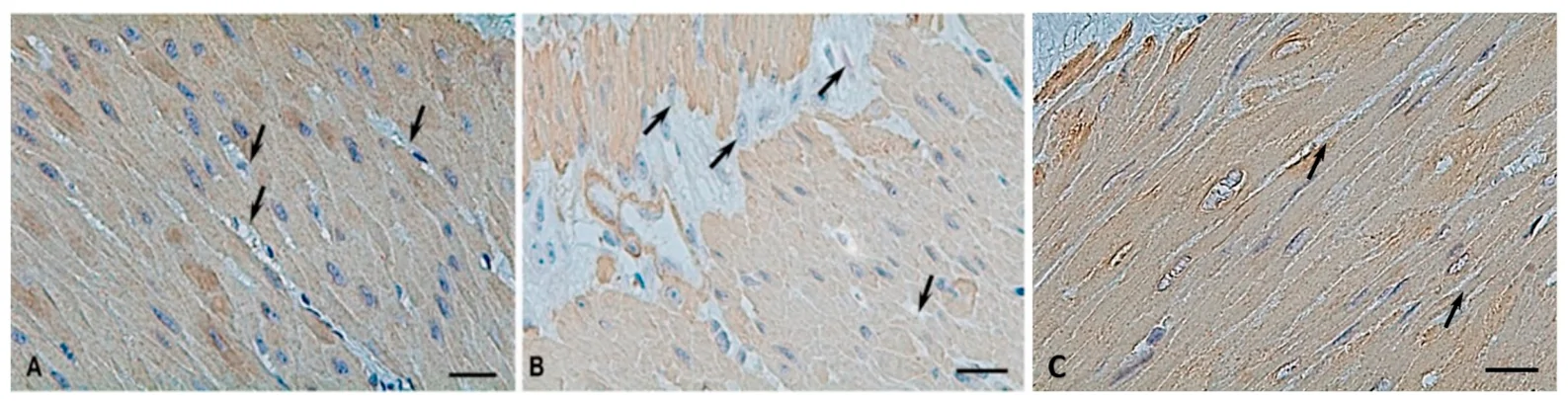
The muscular membrane of the fundal stomach of the Mongolian gerbils. Technique: immunohistochemical detection of α-SMA. (**A**) Vivarium control; (**B**) spaceflight group; (**C**) synchronous experiment group. Note: The arrows indicate: (**A**–**C)** immunonegative areas of the muscle membrane. The scale bar is 10 µm.

## Data Availability

The original contributions presented in this study are included in the article. Further inquiries can be directed to the corresponding author.

## Abbreviations

The following abbreviations are used in this manuscript:

SMT	smooth muscle tissue
ISS	International Space Station
DS	digestive system
SMCs	smooth myocytes cells
α-SMA	alpha-smooth muscle actin
VC	vivarium control
SE	synchronous experiment
SF	spaceflight
